# Multi-*b*-value DWI to evaluate the synergistic antiproliferation and anti-heterogeneity effects of bufalin plus sorafenib in an orthotopic HCC model

**DOI:** 10.1186/s41747-024-00448-y

**Published:** 2024-03-12

**Authors:** Ran Guo, Fang Lu, Jiang Lin, Caixia Fu, Mengxiao Liu, Shuohui Yang

**Affiliations:** 1 Department of Radiology, Shanghai Municipal Hospital of Traditional Chinese Medicine, Shanghai University of Traditional Chinese Medicine, 274 Middle Zhi-jiang Road, Shanghai, 200071 People’s Republic of China; 2 Department of Radiology, Shuguang Hospital Affiliated to Shanghai University of Traditional Chinese Medicine, Shanghai, 201203 People’s Republic of China; 3 Department of Radiology, Zhongshan Hospital, Fudan University, Shanghai, 200032 People’s Republic of China; 4 Shanghai Institute of Medical Imaging, Shanghai, 200032 People’s Republic of China; 5 MR Application Development, Siemens Shenzhen Magnetic Resonance Ltd, Shenzhen, 518057 People’s Republic of China; 6 MR scientific Marketing, Diagnostic Imaging, Siemens Healthineers Ltd, Shanghai, 201318 People’s Republic of China

**Keywords:** Bufalin, Carcinoma (hepatocellular), Diffusion magnetic resonance imaging, Liver neoplasms, Sorafenib

## Abstract

**Background:**

Multi-*b*-value diffusion-weighted imaging (DWI) with different postprocessing models allows for evaluating hepatocellular carcinoma (HCC) proliferation, spatial heterogeneity, and feasibility of treatment strategies. We assessed synergistic effects of bufalin+sorafenib in orthotopic HCC-LM3 xenograft nude mice by using intravoxel incoherent motion (IVIM), diffusion kurtosis imaging (DKI), a stretched exponential model (SEM), and a fractional-order calculus (FROC) model.

**Methods:**

Twenty-four orthotopic HCC-LM3 xenograft mice were divided into bufalin+sorafenib, bufalin, sorafenib treatment groups, and a control group. Multi-*b*-value DWI was performed using a 3-T scanner after 3 weeks’ treatment to obtain true diffusion coefficient D_t_, pseudo-diffusion coefficient D_p_, perfusion fraction *f*, mean diffusivity (MD), mean kurtosis (MK), distributed diffusion coefficient (DDC), heterogeneity index *α*, diffusion coefficient D, fractional order parameter *β*, and microstructural quantity *μ*. Necrotic fraction (NF), standard deviation (SD) of hematoxylin-eosin staining, and microvessel density (MVD) of anti-CD31 staining were evaluated. Correlations of DWI parameters with histopathological results were analyzed, and measurements were compared among four groups.

**Results:**

In the final 22 mice, *f* positively correlated with MVD (*r* = 0.679, *p* = 0.001). Significantly good correlations of MK (*r* = 0.677), *α* (*r* = -0.696), and *β* (*r*= -0.639) with SD were observed (all *p* < 0.010). *f*, MK, MVD, and SD were much lower, while MD, *α*, *β*, and NF were higher in bufalin plus sorafenib group than control group (all *p* < 0.050).

**Conclusion:**

Evaluated by IVIM, DKI, SEM, and FROC, bufalin+sorafenib was found to inhibit tumor proliferation and angiogenesis and reduce spatial heterogeneity in HCC-LM3 models.

**Relevance statement:**

Multi-*b-*value DWI provides potential metrics for evaluating the efficacy of treatment in HCC.

**Key points:**

• Bufalin plus sorafenib combination may increase the effectiveness of HCC therapy.

• Multi-*b*-value DWI depicted HCC proliferation, angiogenesis, and spatial heterogeneity.

• Multi-*b*-value DWI may be a noninvasive method to assess HCC therapeutic efficacy.

**Graphical Abstract:**

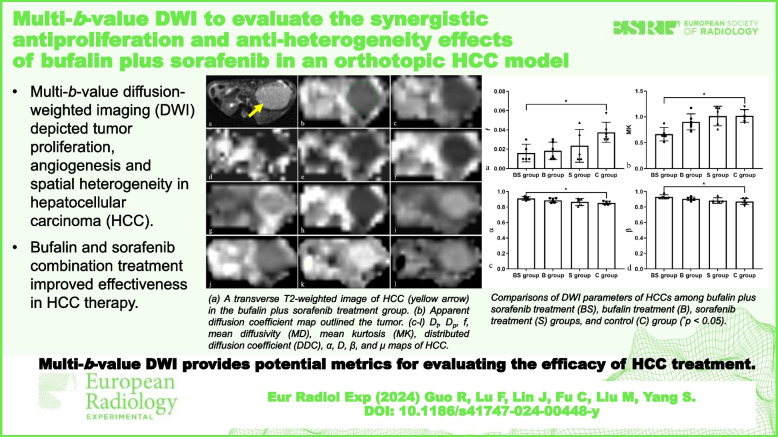

## Background

Hepatocellular carcinoma (HCC) accounts for 75–85% of primary liver cancer cases and is the sixth most common cancer worldwide; moreover, it was the third leading cause of cancer-associated deaths [1]. Because of the high incidence of chronic hepatitis B virus infection, China is a high-risk country for HCC prevalence, which poses a considerable burden on the society [2]. HCC is characterized by aberrant angiogenesis with abundant blood and oxygen supply. Meanwhile, HCC is a high degree heterogeneous cancer [3]. All of the aforementioned causes result in a poor prognosis and an extremely low overall survival rate in HCC patients [4]. Hence, an antiproliferative, antiangiogenetic, and heterogeneity-reducing therapeutic strategy is crucial for patients with HCC, particularly for those with advanced or recurrent disease.

Sorafenib was the only FDA-approved first-line systemic therapy for patients with advanced or unresectable HCC until April 2017 [5]. It is an oral multi-kinase inhibitor, reportedly exerting antiproliferative and antiangiogenic effects on HCC by targeting various growth factor pathways [6]. However, owing to acquired drug resistance, reduced efficacy, and the undesirable side effects of sorafenib, the median overall survival of patients with HCC treated with sorafenib has not significantly improved and it retreated to the second line [7, 8]. Therefore, combination drug treatments of sorafenib with other drugs have gained greatly increasing interest.

Huachansu, a traditional Chinese patent medicine, has been used as a choice for combination drug treatment protocol in HCC—mainly advanced HCC—to obtain stronger and more durable antitumor effects, enhance patients’ quality of life, and prolong progression-free survival [9, 10]. Bufalin is an important component of Huachansu and inhibits tumor proliferation, invasion, and metastasis by influencing several signal pathways such as angiogenesis, apoptosis, and autophagy [11]. Combining bufalin and sorafenib was also observed to produce synergistic antitumoral effects on HCC and lung cancer by suppressing proliferation and inducing apoptosis of tumor cells [12, 13].

Currently, clinical evaluation of HCC angiogenesis, cell proliferation, and tumor parenchyma necrosis involve pathological analysis of specimens obtained through needle biopsy or surgery. Considering their invasiveness and bias of different operators and sampling locations, other robust biomarkers for evaluating HCC are urgently needed. With the development of multi-*b*-value diffusion-weighted imaging (DWI) in magnetic resonance imaging (MRI) and various postprocessing methods, angiogenesis, and cell proliferation within the tumor and cell distribution heterogeneity can be quantified noninvasively by a single MRI scanning [14].

Multi-*b*-value DWI refers to a set of diffusion-weighted raw images obtained by combining different *b-*values and the corresponding excitation numbers. True diffusion coefficient D_t_, pseudo-diffusion coefficient D_p_, and perfusion fraction *f* of intravoxel incoherent motion (IVIM) deriving from a biexponential diffusion model, mean diffusivity (MD) and mean kurtosis (MK) of diffusion kurtosis imaging (DKI) originating from a non-Gaussian diffusion model, and distributed diffusion coefficient (DDC) plus heterogeneity index *α* of a stretched exponential model (SEM) can be used to assess HCC angiogenesis, cellularity, and spatial heterogeneity [15–17]. Fractional-order calculus (FROC), a new non-Gaussian DWI post-processing model, arouses great interest for its potential capability to grade and stage cancer [18]. Diffusion coefficient D, fractional-order derivative in space *β*, and a spatial parameter *μ*, deriving from FROC, can provide additional avenues to assess diffusion process and improve characterization of tissue heterogeneity according to cellular uneven spatial distribution and structural abnormality [18]. So far, only one study in patients with gastrointestinal stromal tumors used FROC model to predict therapeutic response [19], and there has not been an animal study that integrates FROC model with the histopathological evaluation in an orthotopic HCC. Until now, few studies have evaluated angiogenesis, cellularity, and spatial heterogeneity of HCC after a combination therapy with bufalin and sorafenib using both MRI and histopathological investigations.

Therefore, this study uses IVIM, DKI, SEM, and FROC and histopathological approaches to confirm the value of multi-*b*-value DWI for therapeutic effectiveness evaluation and to assess the decelerative effects of angiogenesis, cell proliferation, and spatial heterogeneity by bufalin plus sorafenib therapy in orthotopic HCC-LM3 xenograft mice.

## Methods

### Animal model and treatment

This experiment was approved by the Institutional Animal Care and Use Committee of our hospital. We included 24 male BALB/C nude mice aged 4–6 weeks, weighing 23–25 g (Shanghai Institute of Materia Medica, Chinese Academy of Sciences, Shanghai, China). Animals were housed in specific pathogen-free rooms at constant temperature and humidity and allowed access to food and water ad libitum. Animals were monitored daily for health status to minimize pain and discomfort.

We established orthotopic xenograft models of HCC-LM3 in nude mice for subsequent evaluation. Under aseptic conditions, HCC-LM3 cells (5 × 10^6^/0.2 mL/site) were inoculated subcutaneously in the left axilla of a nude mouse. When the tumor length was > 1 cm, it was removed, cut into 1-mm^3^ blocks, and implanted under the left liver lobe capsules using the tunnel method in 24 nude mice.

At 21 days after tumor implantation, the mice were randomly divided into four groups by using RAND function in Microsoft Excel: bufalin plus sorafenib treatment group (BS group, *n* = 6), bufalin treatment group (B group, *n* = 6), sorafenib treatment group (S group, *n* = 6), and control group (C group, *n* = 6). The mice in the BS group were intraperitoneally injected with 0.2 mL of bufalin solution (Tongtian Bio, Shanghai, China) at a dosage of 20 μg/kg and orally gavaged with 0.2 mL of sorafenib suspension (Bayer Medicine, Leverkusen, Germany) at a dosage of 30 mg/kg daily [6, 20]. The mice in the B group were intraperitoneally injected with 0.2 mL of bufalin solution and orally gavaged with 0.2 mL of 0.9% saline daily. The mice in the S group were intraperitoneally injected with 0.2 mL of 0.9% saline and orally gavaged with 0.2 mL of sorafenib suspension daily. Finally, those in the C group were intraperitoneally injected with 0.2 mL of 0.9% saline and orally gavaged with 0.2 mL of 0.9% saline daily. Treatments were performed from day 1 to 5 of each week, and the animals rested on days 6 and 7 as a cycle for three sequential cycles. At the treatment endpoint, the mortality of mice was assessed.

### MRI protocol

After 21 days of treatment, all nude mice were anesthetized via intraperitoneal injection with a 40 mg/kg dosage of 3% sodium pentobarbital. The mice were then placed into an animal cradle in the prone position. A 3-T MR scanner (MAGNETOM Skyra, Siemens Healthineers, Erlangen, Germany) with an 8-channel animal coil was used to examine the mice. Conventional MRI sequences were performed using fast spin-echo sequences with the following parameters:

- Transverse T2-weighted (time of repetition [TR]/time of echo [TE] 4,000/65 ms; field of view [FOV] 100 mm; flip angle 150°; section thickness 2 mm; intersection gap 0.2 mm; scan time 1:56 min:s);
- Coronal T2-weighted (TR/TE 4,000/70 ms; FOV 100 mm; flip angle 150°; section thickness 2 mm; intersection gap 0.2 mm; scan time 2:28 min:s);
- Transverse T1-weighted (TR/TE 480/11 ms; FOV 100 mm; flip angle 131°; section thickness 2 mm; intersection gap 0.2 mm; scan time 2:4 min:s).

Multi-*b*-value DWI was performed in a transverse plane using a free breathing single-shot echo-planar sequence with the following parameters: TR/TE 3,540/75 ms; separation between 2 diffusion gradient lobes Δ = 42.7 ms; duration of each diffusion gradient δ = 29.4 ms; FOV 75 mm; section thickness 2 mm; intersectional gap 0.4 mm; voxel size 0.6 × 0.6 × 2.0 mm^3^; matrix 120 × 120, bandwidth 463 Hz/pixel; and 11 *b*-values (average number) = 0 (1), 50 (1), 80 (1), 150 (1), 300 (2), 500 (2), 800 (3), 1,000 (3), 1,500 (4), 2,000 (4), and 3,000 (5) s/mm^2^. The autocalibrated partial parallel acquisition technique was used with an acceleration factor of 2.

### Image postprocessing and analysis

The original multi-*b*-value DWI images were imported into an in-house developed postprocessing program based on MATLAB (Mathworks, Natick, MA, USA) for postprocessing. A Gaussian filter with a full width at half maximum of 3 mm was used to suppress the noise of the diffusion images.

IVIM parameter D_t_, D_p_, and *f* maps was obtained via the biexponential signal intensity fitting with 7 *b*-values [21] (0, 50, 80, 150, 300, 500, and 800 s/mm^2^) according to the following formula:

1 $${\text{S}}({\text{b}})/{\text{S}}(0) = (1 - f) \times \mathrm{ exp}(-\mathrm{b }\times {D}_{t}) + f \times \mathrm{ exp}(-\mathrm{b }\times {D}_{p})$$

where S(0) represents the signal intensity without diffusion-weighting, S(b) represents the signal intensity at a particular b.* D*_*t*_ (in 10^-3^ mm^2^/s) represents the true diffusion coefficient reflecting the diffusion of water molecules inside and outside the cell; *D*_*p*_ (in 10^-3^ mm^2^/s) is the pseudo-diffusion coefficient, while *f* represents the perfusion fraction [22].

DKI and SEM parameter maps were derived from 7 *b*-values (0, 500, 800, 1,000, 1,500, 2,000, 3,000 s/mm^2^) using voxel-by-voxel fitting according to the following formulas [15]:

2 $${\text{S}}({\text{b}})/{\text{S}}(0) =\mathrm{ exp}(-\mathrm{b }\times MD + 1/6 \times {{\text{b}}}^{2} \times {MD}^{2} \times MK)$$

3 $${\text{S}}({\text{b}})/{\text{S}}(0) =\mathrm{ exp}\left[{-\left(\mathrm{b }\times DDC\right)}^{\alpha }\right]$$

where *MD* (in 10^-3^ mm^2^/s) is the apparent diffusion coefficient (ADC) after non-Gaussian correction; *MK* is a quantitative index reflecting the degree of deviation of water molecule motion from a Gaussian distribution; *DDC* (in 10^-3^mm^2^/s) reflects the average rate of diffusion; the heterogeneity index *α*, ranging from 0 to 1, describes the heterogeneity of water diffusion [15].

A FROC model was based on the formula (4):

4 $${\text{S}}({\text{b}})/{\text{S}}(0)=\mathrm{ exp }\left[-D \times {\mu }^{2(\beta -1)}{\left(\gamma \times {G}_{d} \times \delta \right)}^{2\beta } \times \left(\Delta -\frac{2\beta -1}{2\beta +1}\times \delta \right)\right]$$

where *G*_*d*_ is the diffusion gradient amplitude, and *δ* and *Δ* are defined earlier. The parameter *D* (in 10^-3^ mm^2^/s) is an attempt to equivalent to conventional ADC;* β* (dimensionless; 0 < *β* ≤ 1) is a spatial fractional order derivative linked to intravoxel tissue heterogeneity, and *μ* (in μm), a spatial parameter, is related to tissue microstructures. These three FROC parameter maps (*D*, *β*, and *μ*) were generated by voxel-by-voxel fitting of the FROC diffusion model with all *b*-values using a Levenberg-Marquardt nonlinear fitting algorithm [19].

**Fig. 1:**
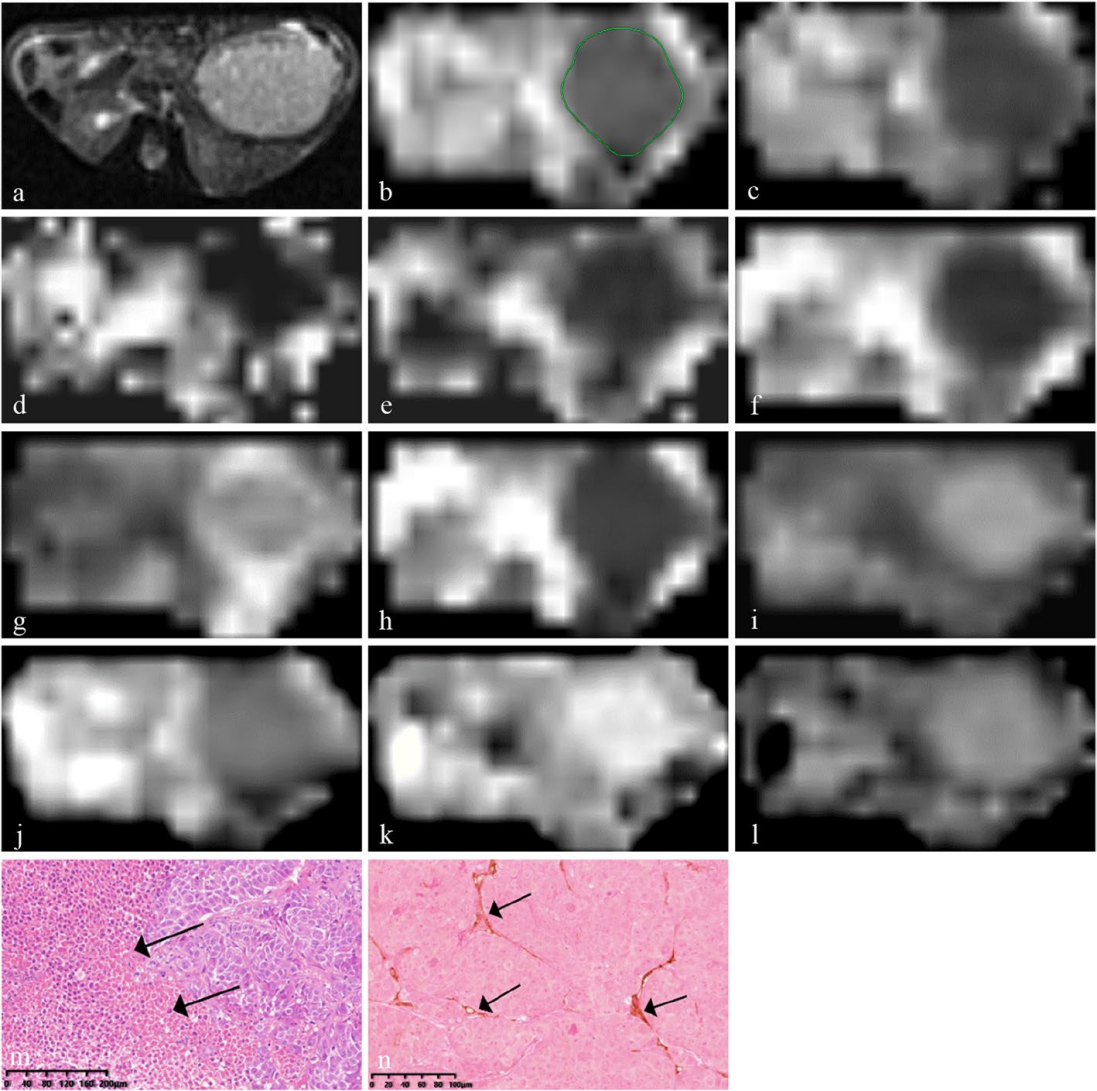
Multi-*b*-value DWI images and corresponding histopathological images of hepatocellular carcinoma in the bufalin plus sorafenib treatment group. **a** A transverse T2-weighted image shows the slice with the maximum tumor diameter. **b** Apparent diffusion coefficient map for outlining the tumor. **c–i** D_t_, D_p_, *f*, mean diffusivity, mean kurtosis, distributed diffusion coefficient, and *α* tumor maps and (**j–l**) D, *β*, and *µ* tumor maps. **m** Hematoxylin-eosin staining showing patchy necrosis (black arrows, ×10). **n** Anti-CD31 staining image showing sparse microvessels (black arrows, ×20)

Further HCC imaging analysis was performed on all the parametric maps. The images of HCC-LM3 mice were evaluated separately by two radiologists with 5 and 19 years of experience in liver imaging. Both the observers were blinded to the groups, treatment, and histopathological results. They reviewed T1W and T2W tumor images to evaluate hemorrhagic foci with significant T1-hyperintensities and T2-hypointensities. The region of interest of each tumor was constructed along the tumor border on the slice with the maximum tumor diameter on ADC map (Fig. 1b). Then, the same region of interest was constructed on the corresponding D_t_, D_p_, *f*, MD, MK, DDC, *α*, D, *β*, and *µ* maps (Fig. 1c–l) [15]. If an apparent hemorrhage existed, the measurement was performed on the nearest above or below slice. Each observer constructed the region of interest separately on each map. Then the average of two measurements obtained from each map were calculated. The same procedure was repeated 4 weeks later to obtain another set of mean values by the same two radiologists. Finally, the mean values from two radiologists’ measurements were averaged to obtain the final results.

### Tumor volume measurement

Tumor volume measurements were performed on T2-weighted transverse images. A radiologist with 5 years of experience in liver imaging used Siemens Syngo via VB10 software to manually construct the tumor boundary on each transverse T2-weighted image slice. Then, the tumor volumes were automatically calculated based on these boundaries.

### Histopathological analysis

The mice were immediately sacrificed after MRI scanning. The tumor and liver tissues were fixed in a 10% buffered formalin solution for > 24 h. According to the slice thickness of each DWI sequence, a central, 2-mm tissue slice was cut and embedded in paraffin. Then, the tumor tissue was cut into 3-μm thin sections for histopathological examination. Hematoxylin-eosin (HE) staining was used to observe tumor morphology, cell proliferation, and necrosis. Anti-CD31 staining was performed to obtain the microvessel density (MVD).

All stained slides were scanned using a KFBIO KF-Pro-120 Digital Pathology Panoramic scanner (Konfoong Biotech International Co., Ltd, Ningbo, China), stored as files with size ranges from 500 to 700 MB, and transferred to a home PC with a screen resolution of 3,840 × 2,160 pixels. K-VIEWER software (Konfoong Biotech International Co., Ltd, Ningbo, China) was used to observe HE and anti-CD31 stained slides. Then, the necrotic fraction (NF), MVD, and the histogram parameter standard deviation (SD) of the distributions of tumor gray pixels were calculated [15]. These analyses were performed by a doctor with 5 years of experience in liver cancer who was blinded to the groups, treatment schedules, and MRI results.

**Fig. 2:**
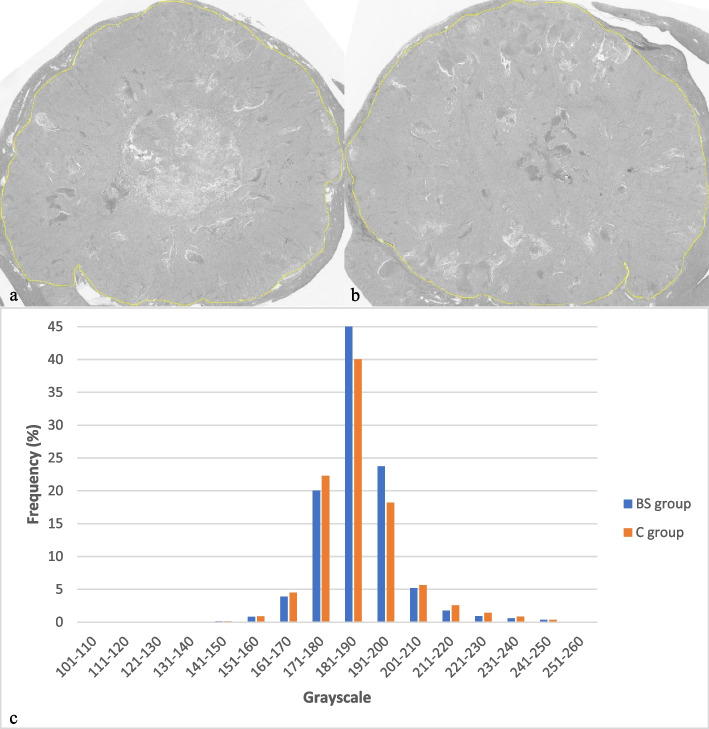
Grayscale images of two whole hematoxylin-eosin -stained slides and corresponding histograms of pixel distribution after treatment. **a** Grayscale image of a tumor in the bufalin plus sorafenib treatment group (histopathological standard deviation 10.475). **b** Grayscale image of a tumor in the control group (histopathological standard deviation 13.795). **c** Histograms of pixel distributions in the grayscale images of the tumors in the bufalin plus sorafenib treatment (blue) and control groups (orange)

First, the digital slides of HE stains were observed under low magnification (× 1 and × 4). Then, five randomly distributed regions were located and observed under high magnification (× 10) (Fig. 1m). The ratio of tumor necrosis area to total tumor area in the FOV, namely NF, was obtained using Image-Pro Plus 6.1 software (Media Cybernetics, Rockville, MD, USA). Second, anti-CD31 stained images were observed under × 1 and × 4 magnifications to locate three regions with the densest CD31 positive vessels. These three regions were magnified by × 20 and MVD was counted using ImageJ v.1.48 software (National Institutes of Health, Bethesda, MD, USA) (Fig. 1n). Finally, the original digital images of HE staining obtained using panoramic scanning were converted to JPG format and then to grayscale images via ImageJ; each pixel of the grayscale image was accompanied by a corresponding gray value (Fig. 2a,b). Histogram analysis of the images was performed to obtain the distribution of gray pixels (Fig. 2c). SD was calculated as an indicator of spatial heterogeneity of each tumor.

### Statistical analysis

SPSS 20.0 software (IBM, New York, USA) was used for all statistical analyses. Measurements are presented as mean ± standard deviation. The Spearman rank correlation test was used to analyze the correlations of DWI parameters with tumor volume, NF, MVD, and SD, respectively. The degree of correlation was determined using the correlation coefficient rho (*r*) that represents the following relationships: 0 ≤ |*r*| < 0.2, poor-to-no relationship; 0.2 ≤ |*r*| ≤ 0.4, fair relationship; 0.4 < |*r*| ≤ 0.6, moderate relationship; 0.6 < |*r*| ≤ 0.8, good relationship; and |*r*| > 0.8, excellent relationship [15]. The Kruskal-Wallis *H* and post hoc Mann-Whitney *U* tests were used to assess differences in DWI parameters, volume, NF, MVD, and SD among the four groups. The intra- and interobserver agreement of DWI parameters were evaluated by using intraclass correlation coefficients (ICCs). ICCs were defined as follows: ≤ 0.40, poor-to-fair reliability; 0.41–0.60, moderate reliability; 0.61–0.80, good reliability; and > 0.80, excellent reliability [15]. A two-tailed *p* value of < 0.050 was considered statistically significant.

## Results

### Mortality and scan success rate of HCC-LM3 models

During the treatment period, one mouse died of diffuse peritoneal metastasis in the S group (1/6).

All MR scans were successfully performed on the surviving 23 mice, and the obtained images had no obvious artifacts, which could be used for further imaging evaluation. Except for one mouse in the BS group that did not develop a tumor, all 22 HCC tumors clearly showed slightly hypointense and hyperintense on T1W and T2W images, respectively. Intratumoral hemorrhages were observed in two tumors in the B group, one in the S group, and one in the C group, but none were located in the maximum diameter slices of the tumor.

### Correlations between DWI and histopathological results in HCC-LM3 models

In the final 22 mice, no significant correlations were observed between DWI parameters and tumor volume (all *p* > 0.050). D_t_ and *µ* were moderately and positively correlated with NF (*r* = 0.484, *p* = 0.022; *r* = 0.424, *p* = 0.049). *f*, MD, MK, DDC, *α*, D, and *β* showed moderate to good correlations with MVD (all *p* < 0.050) (Table 1). D_t_, MD, and DDC were found moderately and negatively correlated with SD (*r* = -0.538, *p* = 0.010; *r* = -0.586, *p* = 0.004; *r* = -0.581, *p* = 0.005). Significantly good and positive correlation of MK with SD (*r* = 0.677, *p* = 0.001), and significantly good and negative correlations of *α* and *β* with SD (*r* = -0.696, *p* < 0.001; *r* = -0.639, *p* = 0.001) were observed (Table 1).

**Table 1:** Correlations between diffusion-weighted imaging and histopathological results in 22 tumor models

	Volume		Necrotic fraction		Microvessel density		Standard deviation	
	*r*	*p*	*r*	*p*	*r*	*p*	*r*	*p*
D_t_	-0.121	0.593	0.484	**0.022**	-0.313	0.156	-0.538	**0.010**
D_p_	-0.207	0.356	-0.383	0.078	0.277	0.213	0.421	0.051
*f*	-0.047	0.836	-0.315	0.153	0.679	**0.001**	0.411	0.057
MD	0.054	0.812	0.335	0.128	-0.508	**0.016**	-0.586	**0.004**
MK	0.105	0.643	-0.299	0.176	0.630	**0.002**	0.677	**0.001**
DDC	0.124	0.584	0.302	0.173	-0.475	**0.026**	-0.581	**0.005**
*α*	-0.263	0.238	0.229	0.305	-0.590	**0.004**	-0.696	**< 0.001**
D	-0.231	0.301	0.117	0.603	-0.468	**0.028**	-0.239	0.284
*β*	-0.160	0.476	0.392	0.071	-0.629	**0.002**	-0.639	**0.001**
*µ*	-0.345	0.116	0.424	**0.049**	-0.137	0.542	-0.418	0.053

Significant results reported in bold characters. *DDC* Distributed diffusion coefficient*, MD* Mean diffusivity*, MK* Mean kurtosis

### Comparisons of DWI parameters among four HCC-LM3 groups

**Fig. 3:**
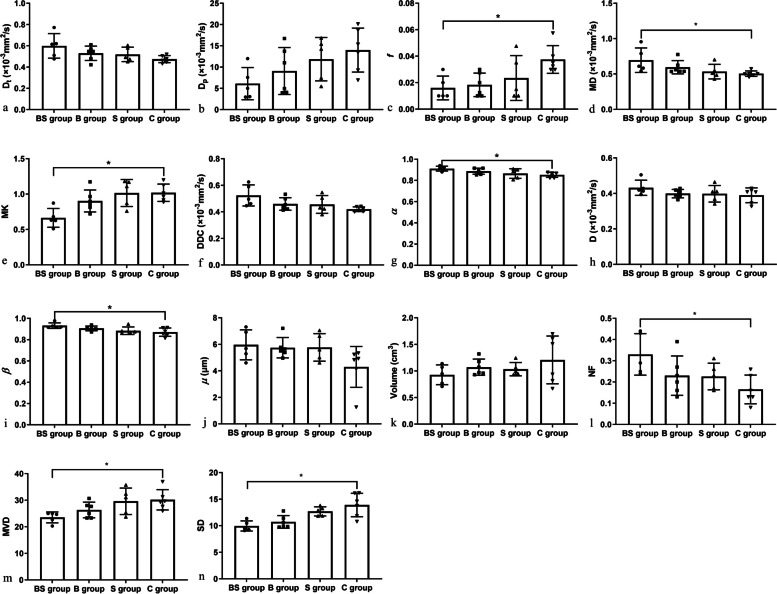
Comparisons of diffusion-weighted imaging and histopathological parameters of hepatocellular carcinoma LM3 models among the four groups. The results are expressed as the mean ± standard deviation (^*^p < 0.05). *B* Bufalin treatment, *BS* Bufalin plus sorafenib treatment,* C* Control, *DDC* Distributed diffusion coefficient, *MD* Mean diffusivity, *MK* Mean kurtosis, *MVD* Microvessel density, *NF* Necrotic fraction, *S* Sorafenib treatment, *SD* Histopathological standard deviation

Significant differences were observed in *f*, MD, MK, *α*, and *β* among the four groups (all *p* < 0.050) (Table 2). However, no significant differences were found in other DWI parameters (all *p* > 0.050) (Table 2, Fig. 3). Furthermore, significantly lower *f* and MK, and significantly higher MD, *α*, and *β* in the BS group than those in the C group were demonstrated by the post hoc Mann-Whitney *U* test with Bonferroni adjustment (all *p* < 0.050) (Table 4, Fig. 3), while no significant inter-group differences of *f*, MD, MK, *α*, and *β* were observed between other groups (all *p* > 0.050).

**Table 2:** Comparisons of diffusion-weighted imaging parameters among four groups

**Parameters (mean ± SD)**	**BS group (*n* = 5)**	**B group (*n* = 6)**	**S group (*n* = 5)**	**C group (*n* = 6)**	***χ***^***2***^	***p***
**D**_**t**_**(×10**^**-3**^** mm**^**2**^**/s)**	0.599 ± 0.116	0.530 ± 0.067	0.519 ± 0.068	0.475 ± 0.034	5.739	0.125
**D**_**p**_**(×10**^**-3**^** mm**^**2**^**/s)**	6.114 ± 3.782	9.071 ± 5.513	11.848 ± 5.092	13.988 ± 5.174	6.580	0.087
***f***	0.016 ± 0.009	0.018 ± 0.009	0.024 ± 0.017	0.038 ± 0.010	8.910	**0.031**
**MD(×10**^**-3**^** mm**^**2**^**/s)**	0.695 ± 0.173	0.596 ± 0.094	0.534 ± 0.103	0.505 ± 0.038	9.502	**0.023**
**MK**	0.665 ± 0.132	0.903 ± 0.155	1.016 ± 0.191	1.020 ± 0.123	10.375	**0.016**
**DDC(×10**^**-3**^** mm**^**2**^**/s)**	0.524 ± 0.079	0.459 ± 0.047	0.456 ± 0.067	0.420 ± 0.018	7.523	0.057
***α***	0.912 ± 0.023	0.887 ± 0.028	0.865 ± 0.046	0.853 ± 0.023	9.002	**0.029**
**D(×10**^**-3**^** mm**^**2**^**/s)**	0.432 ± 0.043	0.399 ± 0.024	0.398 ± 0.047	0.390 ± 0.042	3.169	0.366
***β***	0.932 ± 0.026	0.906 ± 0.022	0.884 ± 0.036	0.871 ± 0.039	9.739	**0.021**
***µ*(μm)**	5.962 ± 1.130	5.745 ± 0.766	5.766 ± 1.035	4.297 ± 1.540	6.795	0.079

Significant results reported in bold characters. Data are expressed as mean ± SD. *B* Bufalin treatment, *BS* Bufalin plus sorafenib treatment, *C* Control, *DDC* Distributed diffusion coefficient, *MD* Mean diffusivity, *MK* Mean kurtosis, *S* Sorafenib treatment, *SD* Standard deviation

### Comparisons of histopathological results among four HCC-LM3 groups

Histopathological NF, MVD, and SD were significantly different among the four groups (all *p* < 0.050) (Table 3). Further post hoc Mann-Whitney *U* test with Bonferroni adjustment showed that MVD and SD were significantly lower, while NF were significantly higher in the BS group than those in the C group (all *p* < 0.050) (Table 4, Fig. 3). However, no significant inter-group differences of NF, MVD, and SD were observed between other groups (all *p* > 0.050). Although no significant differences were found among four groups (all *p* > 0.050), tumor volumes in the three treatment groups were all slightly smaller than those in the control group. Furthermore, tumor volumes in the BS group were slightly smaller than those in the B and S groups (all *p* > 0.050) (Table 3, Fig. 3).

**Table 3:** Comparisons of histopathological results among four groups

**Parameters (mean ± SD)**	**BS group (*n* = 5)**	**B group (*n* = 6)**	**S group (*n* = 5)**	**C group (*n* = 6)**	***χ***^***2***^	***p***
**Volume (cm**^**3**^**)**	0.928 ± 0.186	1.072 ± 0.153	1.034 ± 0.125	1.208 ± 0.449	1.263	0.738
**Necrotic fraction**	0.330 ± 0.098	0.230 ± 0.093	0.226 ± 0.063	0.165 ± 0.068	7.965	**0.047**
**Microvessel density**	23.533 ± 2.036	26.333 ± 2.936	29.600 ± 4.991	30.167 ± 3.811	9.545	**0.023**
**Histopathological SD**	9.958 ± 0.945	10.719 ± 1.195	12.701 ± 0.866	13.901 ± 2.212	13.972	**0.003**

Significant results reported in bold characters. Data are expressed as mean ± SD.* B* Bufalin treatment, *BS* Bufalin plus sorafenib treatment,* C* control,* S* Sorafenib treatment, *SD* Standard deviation

**Table 4:** Comparisons of diffusion-weighted imaging and histopathological results among four groups

Parameters	Groups											
	(BS)-(B)		(BS)-(S)		(BS)-(C)		(B)-(S)		(B)-(C)		(S)-(C)	
	*Z*	*p*^*†*^	*Z*	*p*^*†*^	*Z*	*p*^*†*^	*Z*	*p*^*†*^	*Z*	*p*^*†*^	*Z*	*p*^*†*^
Necrotic fraction	-1.685	0.552	-1.393	0.982	-2.812	**0.030**	-0.230	1.000	-1.182	1.000	-1.357	1.000
Microvessel density	1.210	1.000	2.390	0.101	2.781	**0.033**	-1.286	1.000	1.648	0.597	0.284	1.000
Histopathological SD	0.907	1.000	2.581	0.059	3.281	**0.006**	-1.789	0.442	2.490	0.077	0.585	1.000
*f*	0.372	1.000	0.968	1.000	2.684	**0.044**	-0.640	1.000	2.425	0.092	1.673	0.566
Mean diffusivity	-1.000	1.000	-2.143	0.193	-2.823	**0.029**	1.238	1.000	-1.912	0.336	-0.585	1.000
Mean kurtosis	1.780	0.450	2.532	0.068	3.009	**0.016**	-0.865	1.000	1.289	1.000	0.365	1.000
*α*	-1.152	1.000	-1.929	0.322	-2.873	**0.024**	0.863	1.000	-1.806	0.426	-0.859	1.000
*β*	-1.500	0.801	-2.240	0.150	-2.984	**0.017**	0.839	1.000	-1.556	0.718	-0.644	1.000

Significant results reported in bold characters. *p*^*†*^ Mann-Whitney *U* test with Bonferroni adjustment. *B* Bufalin treatment, *BS* Bufalin plus sorafenib treatment,* C* Control,* S* Sorafenib treatment, *SD* Standard deviation

### Intra- and interobserver agreement on MRI parameters in HCC-LM3 models

The intra- and interobserver agreements of all DWI parameter measurements were excellent (Table 5).

**Table 5:** Intra- and interobserver agreement of diffusion-weighted imaging parameters in 22 tumor models

	Intraobserver^a^			Interobserver		
	ICC	95% CI	*p*	ICC	95% CI	*p*
D_t_	0.987	0.969–0.995	< 0.001	0.990	0.975–0.996	< 0.001
D_p_	0.989	0.974–0.995	< 0.001	0.978	0.943–0.991	< 0.001
*f*	0.945	0.871–0.977	< 0.001	0.937	0.854–0.973	< 0.001
Mean diffusivity	0.992	0.979–0.997	< 0.001	0.990	0.977–0.996	< 0.001
Mean kurtosis	0.984	0.962–0.993	< 0.001	0.983	0.959–0.993	< 0.001
Distributed diffusion coefficient	0.986	0.966–0.994	< 0.001	0.983	0.957–0.993	< 0.001
*α*	0.973	0.936–0.988	< 0.001	0.966	0.922–0.986	< 0.001
D	0.988	0.970–0.995	< 0.001	0.981	0.955–0.992	< 0.001
*β*	0.984	0.962–0.993	< 0.001	0.976	0.944–0.990	< 0.001
*µ*	0.979	0.950–0.991	< 0.001	0.930	0.842–0.970	< 0.001

^a^A radiologist with 19 years of experience in liver magnetic resonance imaging interpretation. *CI* Confidence interval, *ICC* Intraclass correlation coefficient

## Discussion

Through *in vivo* multi-*b*-value DWI and postprocessing of a biexponential IVIM, non-Gaussian distribution models of DKI, SEM, and FROC, parameter MK of all tumor-bearing nude mice were found positively correlated with SD, while D_t_, MD, DDC, *α*, and *β* were negatively correlated with SD. Besides, correlations of D_t_ and *µ* with NF, and *f*, MD, MK, DDC, *α*, D, and *β* with MVD were observed. We also found that *f*, MK, MVD, and SD were significantly decreased, while MD, *α*, *β*, and NF were significantly increased in the BS group compared with those in the C group.

DWI-derived parameters, D_t_, MD, DDC, and D, reflect the diffusion of water molecules and represent the extent of cell proliferation and tumor necrosis in HCCs [15, 17, 23]. These have been demonstrated in several studies of gastrointestinal stromal tumors and HCC, where D_t_, MD, DDC, and D were much higher following therapy, presumably due to necrosis [15, 17, 19, 24]. The current study also demonstrated that NF was increased in the BS group compared with the C group, meanwhile, MD was higher in the BS group and positive correlation between D_t_ and NF was found. The possible reasons are as follows: first, combination treatment enhanced antiproliferation and antiangiogenesis effects on HCC and induced pronounced necrosis in the tumor; second, decreased tumor cell density and increased extracellular spaces after treatment also led to unrestricted diffusion of water molecular.

We failed to observe other significant correlations of MD, DDC, and D with NF. However, moderate and negative correlations between D_t_, MD, DDC, and SD were discovered, which may be explained as follows. First, the relatively short period effect on HCC, along with small sample size and drug resistance of sorafenib prevented more meaningful correlations between water molecular diffusion parameters and NF. Second, before apparent necrosis was discovered to significantly increase diffusion parameters, early diminished cellularity and patchy necrosis might have a notable influence on tumor heterogeneity.

These results are somewhat in line with a treatment prediction study in gastrointestinal stromal tumor, which indicated that D from FROC showed no significant difference between the good responder and poor responder groups in early stage as early as 2 weeks, while the percentage change in D was already higher in the good responder group [19]. *µ* represents the mean free diffusion length and relates to D [18]; although was moderately correlated with NF in this study, its *p* value was relatively high at 0.049. Due to the inadequate information of *µ* available from earlier research in tumors, its value is still uncertain [25]. *µ* was found significantly lower in non-muscle-invasive bladder cancer than muscle-invasive bladder cancer and in the high- than low-grade bladder cancer [18], while no differences between benign prostatic hyperplasia and prostate cancer, or between Cytokeratin 19-positive and Cytokeratin 19-negative HCCs, were observed [23, 26]. Future robust studies with different *b*-value settings and postprocessing models might worth further exploring.

D_p_ and *f* reflected microcirculatory perfusion [22], and were found positively correlated with angiogenic factors [27]. However, due to the interference of tissue structure, tortuous vascularity, particle or gland excretion, and different components, such as capillaries that cannot reflect the macroscopic tumors and vessels [28], it is challenging for D_p_ and *f* to reliably depict tumor cell proliferation in studies of endometrial carcinoma and lung adenocarcinoma [22, 29]. Our study in HCCs showed good and positive correlation between *f* and MVD, which demonstrated the value of *f* for accurate reflection of tumor microcirculation. No correlation was found between D_p_ and MVD. This might be attributed to inherent poor signal-to-noise ratio of D_p_ and subsequently low precision in measurement [21]. Furthermore, correlations between MVD and some non-Gaussian distribution parameters indicated that effectively synergistic treatment-related MVD reduction might relate to reduced cellularity and tumor heterogeneity [30, 31].

MK, *α*, and *β* provide information about complexity and irregularity of tissue components and quantify heterogeneity as the reflection of the complicated tumor microstructure [15, 18, 25]; therefore, these parameters could be used to capture the dynamic process of spatial heterogeneity changes in HCC. Positive correlation of MK with pathological grade of HCC was commonly accepted [32]. MK was significantly lower in completely necrotic HCCs than viable HCCs, demonstrating a new possibility for evaluating HCC treatment, as viable HCCs contain higher cellularity and atypia, more vascular hyperplasia, and necrosis, exhibiting higher structural complexity [33]. Similarly, previous studies showed that *α* and *β* are inversely proportional to tissue heterogeneity [34, 35]. Our previous study demonstrated that high MK and low *α* correlated with high SD in an orthotopic xenograft HCC model [15]. Good correlations of MK, *α*, and *β* with SD in this study confirmed that tumor with low MK and high *α* values had a small degree of heterogeneity, and indicated that high *β* values might be the analogous indicator as *α*. Meanwhile, the lower heterogeneity reflected by lower MK, higher *α*, and higher *β* values were also observed after combination treatment. Similar results were found in other studies in different tumors with consistent drops in MK and rises in *α* and *β* following antitumor treatment, indicating that the therapy reduced the tumor’s heterogeneity [19, 24, 36]. Correspondingly, HCCs have intrinsically variable morphologies, immunological phenotypes, and gene mutational statuses in histopathology [3], which may also show various clusters, exhibit geographic heterogeneity, or alter dynamically over time or in response to therapy [3, 37]. Considering that conventional ADC demonstrated insufficient efficacy in HCC evaluation [16], and non-Gaussian diffusion parameters MD, DDC, and D, suggesting mainly tumor necrosis, showed limited values in our early necrosis evaluation after treatment, the advanced imaging parameters such as MK, *α*, and *β* might help to depict tumor components’ heterogeneity and spatial distribution changes after treatment earlier and better. Until now, no study had investigated the correlations between FROC models (D, *β*, and *µ*) and SD. Furthermore, considering the limitations of the conventional therapy response evaluation criteria mainly focusing on the long diameter of the solid tumor [38], different DWI models might help to provide additional effective assessment in necrosis, vascularization, and heterogeneity. A consistent result was found in a gastrointestinal stromal tumor after second-line sunitinib therapy, the change in D at 2 weeks significantly outperformed tumor diameter change in response prediction [19]. In our study, it was also demonstrated that MD, which represented the pure water molecular diffusion, increased following the synergistic treatment. Meanwhile, MK, *α*, and *β*, reflecting heterogeneity, changed earlier than the tumor shrinking, which might associate with disease prognosis [39].

Given that limited improvement in survival and prognosis of patients with advanced HCC treated with sorafenib, combination drug treatments to circumvent resistance by increasing tumor cell sensitivity and overcoming toxicity might help extend the survival [7, 12]. In our study, five orthotopic HCC-LM3 mice survived in the BS group, and the other one did not develop into an isolated tumor. Recent researches shown that the treatment of HCC with a combination of bufalin and sorafenib can reverse HCC resistance to sorafenib, enhance antiproliferative and antiangiogenesis effects synergistically, and induce HCC apoptosis and tumor necrosis [12, 23, 40]. Even without a substantial decrease in tumor volume, MK, MVD, and SD were much lower and NF, *α*, and *β* were significantly higher in our BS group than those in the C group. These results revealed that bufalin and sorafenib may have already suppressed early-stage HCC tumor growth and reduced tumor spatial heterogeneity before substantial decrease of tumor volume appeared [19]. The antiangiogenic effects of bufalin plus sorafenib restricted the tumor blood supply and potentially increased tumor cell death and parenchymal necrosis [40].

DWI parameters, tumor volume, and histopathological NF, MVD, and SD in the S groups were not significantly different from those in the C group, which may be attributable to sorafenib resistance on HCC cell proliferation, angiogenesis, autophagy, and epithelial-mesenchymal transition [7, 41]. Consistent with the finding of a previous study on HCC, which showed that incompletely and nonuniformly distributed necrosis within tumor could increase tumor spatial heterogeneity, HCC cells that poorly responded to sorafenib may result in uneven cell death and parenchymal necrosis within tumors in the S group and reduce the differences of cell proliferation, necrosis, and spatial heterogeneity between the S and C group [15]. Although no significant differences of MVD, NF, and SD between BS and S group were demonstrated in this study, compared to the S group, the tendencies of low MVD and SD, and high NF in the BS group confirmed the synergistic effects by inducing tumor necrosis, reducing tumor spatial heterogeneity, and bringing about the antiangiogenic efficacy. The results of a previous study examining the combination treatment of ShuangDan capsules (a Chinese patent medicine) plus sorafenib in a HepG2 xenograft model showed that the combined therapy exhibited superior effects against HCC than sorafenib alone [42]. In this study, although no significant differences of DWI parameters were observed between the B, S, and C groups, *α* and *β* were slightly higher, while MK and SD were slightly lower in the B group than in the S and C groups. Hence, further study with large sample size concerning mechanisms of bufalin on HCC might help to give the explanations.

This study has several limitations. First, the histopathological sections and measured DWI layers may not have completely matched. Furthermore, the slice histopathological SD might not fully represent the spatial heterogeneity of the whole tumor. Future studies with large sample size to validate these discoveries are needed. Second, semiautomatic MVD calculation based on histopathological anti-CD31 staining could produce offsets; software analyses should increase the reliability of these results in the future. Third, limited by the signal-to-noise ratio (SNR) and tumor size, the highest *b*-value used in this study was 3,000 s/mm^2^, more combinations of *b*-values might further improve the feasibility and reliability for non-Gaussian models. Finally, even if the implanted HCC cell lines were the same, human and animal HCC models are not completely consistent; the therapeutic effects of bufalin plus sorafenib may be different between mice and humans. Thus, clinical trials are needed.

In conclusion, our multi-*b*-value DWI study showed that bufalin plus sorafenib inhibited angiogenesis, cell proliferation, and reduced tumor spatial heterogeneity in an orthotopic HCC-LM3 xenograft model. The combination of bufalin and sorafenib should be further examined as potential anticancer therapy. Future studies may use multi-*b*-value DWI to evaluate the efficacy of combination drug treatment.

## Data Availability

The datasets used and/or analyzed during the current study are available from the corresponding author on reasonable request.

## Abbreviations

ADC: Apparent diffusion coefficient
DDC: Distributed diffusion coefficient
DKI: Diffusion kurtosis imaging
DWI: Diffusion-weighted imaging
FOV: Field of view
FROC: Fractional-order calculus
HCC: Hepatocellular carcinoma
HE: Hematoxylin-eosin
IVIM: Intravoxel incoherent motion
MD: Mean diffusivity
MK: Mean kurtosis
MRI: Magnetic resonance imaging
NF: Necrotic fraction
SD: Standard deviation
SEM: Stretched exponential model
TE: Time of echo
TR: Time of repetition
